# Frequency of Type-2 diabetes mellitus in Nephropathic patients and comparison of mean magnesium levels in Nephropathic patients with and without Type-2 diabetes mellitus

**DOI:** 10.12669/pjms.335.12974

**Published:** 2017

**Authors:** Mariam Azeem, Adil Iqbal, Nasir Farooq Butt, Fawad Ahmad Randhawa, Uzma Malik

**Affiliations:** 1 Mariam Azeem, Department of Medicine, Mayo Hospital, Lahore, Pakistan; 2 Adil Iqbal, Department of Medicine, Mayo Hospital, Lahore, Pakistan; 3 Nasir Farooq Butt, Department of Medicine, Mayo Hospital, Lahore, Pakistan; 4 Fawad Ahmad Randhawa, Department of Medicine, Mayo Hospital, Lahore, Pakistan; 5 Uzma Malik, Department of Medicine, Mayo Hospital, Lahore, Pakistan

**Keywords:** Diabetes mellitus, Frequency, Mean magnesium levels, Nephropathy

## Abstract

**Objective:**

The objective of this study was to determine the frequency of Type 2 diabetes mellitus (T2DM) in patients with nephropathy (i.e. Chronic Kidney Disease Stage 1 to 3) and to compare the mean magnesium levels in diabetic nephropathic patients and non-diabetic nephropathic patients

**Methods:**

This cross-sectional study was conducted in department of Medicine, Mayo Hospital Lahore from August 2014 to February 2015. Using non-probability purposive sampling 200 nephropathic (Chronic Kidney Disease Stage 1 to 3) patients were selected. Patients were assessed for T2DM and divided in two groups on the basis of presence or absence of DM. Magnesium levels were recorded in both groups. Percentages, mean, standard deviation and unpaired t-test was used to assess the data. SPSS was used for analysis of information.

**Results:**

Total number of cases were 200, 43.5% (n=87) out of them were between 25-50 years of age while 56.5% (n=113) patients were between 51-70 years. The Mean+SD was calculated to be 51.38+11.51 years. The male patients were 48.5 %(n=109) while 51.5 %(n=91) were females. The frequency of DM in patients with nephropathy was 25.5% (n=51). Comparison of mean magnesium levels in nephropathic patients with and without diabetes was done. The results showed nephropathic patients having diabetes had 1.54+0.301 mg/dL magnesium levels while cases without diabetes had 1.92+0.313 mg/dL levels of magnesium, p value was calculated as 0.001 showing a significant difference between the two groups.

**Conclusion:**

The frequency of diabetes mellitus is higher among patients with nephropathy while on comparison of mean magnesium levels, nephropathic patients with diabetes had significant lower levels of magnesium as compared to without diabetes.

## INTRODUCTION

Diabetes mellitus (DM) is an important cause of morbidity and mortality worldwide.^1^ Globally, in 2014, there were 422 million adults having diabetes. By 2035 this will rise to 642 million.^2^ Type-2 DM is associated with micro vascular and macro vascular complications.^1^ DM is the most common cause of nephropathy. Diabetic nephropathy (DN) is increasing rapidly around the globe and is considered as one of the major micro vascular complication. Nephropathy, characterized by proteinuria, is one of the most serious long-term complications of DM. The proportion of DN is increasing worldwide. DN is the leading cause of chronic kidney diseases and end-stage renal disease, which constitutes the major workload of dialysis centers worldwide.^3^

Magnesium (Mg) is an important mineral which is used as a cofactor in many metabolic reactions of the body. Lower intake of Mg and low serum Mg concentrations are associated with metabolic syndrome, insulin resistance, and Type-2 diabetes.^4^ The normal adult value for Mg is 1.7–2.4 mg/dL (1.5–2.4 mEq/L; 0.7–1 mmol/L). Hypomagnesemia can be clinically defined as a concentration of serum Mg ≤1.6 mg/dL or 0.66 mmol/L or ≤2SD below the mean of the general population.^5^ Hypomagnesaemia has been reported to occur among patients of DM.^6^ This may increase the risk of cardiovascular abnormalities and also has a strong association with diabetic nephropathy.^7^,^8^

Approximately 1/3^rd^ of Type-2 diabetes patients have hypomagnesemia due to enhanced renal excretion. The relation between Type-2 diabetes mellitus and Mg deficiency is yet to be elaborated completely.^9^ An overt clinical hypomagnesemia or a chronic latent Mg deficit is seen in patients with Type-2 diabetes, especially in those in which glycemic profile is poorly controlled.^10^ Dewitte K et al. showed that mean magnesium level in patients with diabetic nephropathy was 0.489±0.05 while in non-diabetic group with kidney disease this was 0.534±0.05 (unpaired t-test: P<0.001).^11^ Type-2 diabetes mellitus can cause low serum Mg levels resulting in worsen glycemic control in diabetes patients, this develops a cycle that increases the risk of chronic macro-vascular and micro-vascular diabetic complications.^12^ The findings by Prabodh S et al suggested that hypomagnesaemia can be associated with development of diabetic nephropathy. The mean magnesium levels of cases (1.60±0.32 mEq/L) were markedly lower than the control groups 2.14±0.16 mEq/L (p<0.05).^8^

The rationale of the study was to evaluate frequency of diabetes mellitus in patients presenting with nephropathy and to compare the mean magnesium levels in nephropathic patients with and without diabetes so that this easily available, cost effective test may be used as a predictor for the development of nephropathy and progression to End stage renal disease (ESRD) in Type-2 diabetic patients. This study was intended to give timely treatment to control magnesium level and delay the development of nephropathy if we find decreased mean magnesium levels in Type-2 diabetic nephropathic patients.

## METHODS

It was a Cross sectional study done in West Medical Mayo Hospital Lahore from August 2014 to February 2015. A total of 200 patients were included in the study. Non-probability, purposive sampling was done. The inclusion criteria comprised of the patients 25-70 years old irrespective of their genders, nephropathic patients i.e. National Kidney Foundation Chronic Kidney Disease (CKD) Stage 1 to 3 and having proteinuria i.e. urinary protein > 30 mg per 24 hours.

Patients taking medication like steroids and beta agonists, on hemodialysis (CKD Stage 4 and 5), taking magnesium supplementation or being treated with drugs known to modify magnesium metabolism, history of cardiovascular incidence (Coronary artery disease e.g. Acute coronary syndrome) on medical record or critically ill i.e. in intensive care unit (ICU), and pregnant females were excluded.

All nephropathic patients were divided in two groups by virtue of presence or absence of Diabetes mellitus i.e. Group 1: Diabetic Nephropathic Patients, Group 2: Non-diabetic Nephropathic patients. Demographic information of the patients was also obtained. Diabetic status and Magnesium levels were recorded.

Data was entered in SPSS version 23 and analyzed through it. Descriptive statistics was calculated as per type of data (quantitative or qualitative). Mean ± Standard Deviation for quantitative variables such as age and Magnesium level in nephropathic patients with and without diabetes was calculated. For qualitative variables such as gender and diabetes, frequency and percentages were used. Independent sample T-test was applied for comparison of mean magnesium level. P-value of ≤ 0.05 was considered significant.

## RESULTS

After taking approval from Institutional Review Board (IRB), a total of 200 cases that fulfilled the inclusion criteria were included in the study to determine the frequency of diabetes mellitus in nephropathic patients and to compare mean magnesium levels in nephropathic patients with and without diabetes.

Age distribution of the patients showed that 43.5% (n=87) were between 25-50 years of age while 56.5% (n=113) were between 51-70 years of age, Mean ± SD was calculated as 51.38±11.51 years. Patients were distributed according to gender which shows that 48.5% (n=109) were male while 51.5% (n=91) were females. Among the causes of nephropathy, DM was the most common cause i.e. frequency of diabetes mellitus was 25.5% (n=51) (Table-I). The second prevalent cause of nephropathy was Hypertension (25%, n=50). Other causes included use of Medications (e.g. nonsteroidal anti-inflammatory drugs, Hakeem/Desi, etc.), glomerulonephritis, obstructive uropathy, Chronic pyelonephritis, Toxic nephropathy and unknown)

**Table-I:** Frequency of diabetes mellitus in patients with nephropathy (n=200).

*Diabetes mellitus*	*No. of patients with nephropathy*	*%*
Yes	51	25.5
No	149	74.5

Total	200	100

Multiple linear regression was used to see the influence of various variables and results are shown in Table-II. Analysis of the two groups at baseline is shown in Table-III.

**Table-II:** Multiple Linear Regressions.

*Coefficients^a^*									

*Model*		*Unstandardized Coefficients*		*Standardized Coefficients*	*t*	*Sig.*	*Correlations*		
	
		*B*	*Std. Error*	*Beta*			*Zero-order*	*Partial*	*Part*
1	(Constant)	1.556	0.344		4.519	0.000			
	Age	-0.003	0.003	-0.113	-1.212	0.227	-0.141	-0.087	-0.076
	Diabetes Mellitus	0.365	0.052	0.453	7.079	0.000	0.475	0.454	0.442
	GFR	0.001	0.003	0.022	0.191	0.849	0.169	0.014	0.012
	Serum Creatinine	-0.099	0.132	-0.075	-0.753	0.452	-0.134	-0.054	-0.047
	Weight	-0.001	0.003	-0.039	-0.427	0.670	-0.035	-0.031	-0.027
	Proteinuria	7.064E-5	0.000	0.027	0.425	0.671	0.006	0.031	0.027

a Dependent Variable: Magnesium.

**Table-III:** Analysis of the two groups at baseline.

	*Group 1 (Diabetic Nephropathic Patients)*	*Group 2 (Non-diabetic Nephropathic Patients)*
Age	52.75 ± 10.7	50.93 ± 11.7
Weight	74.35 ± 10.77	72.9 ± 9.5
Serum Creatinine	1.41 ± 0.38	1.28 ± 0.32
GFR	60.96 ± 20.8	69.6 ± 21.17
Proteinuria	280.56 ± 144.22	268.85 ± 130.40

Comparison of mean magnesium levels in nephropathic patients with and without diabetes showed patients with diabetes mellitus and nephropathy had 1.54±0.301 mg/dL magnesium levels while cases without diabetes had 1.92±0.313 mg/dL levels of magnesium. P value was calculated as 0.001 showing a significant difference between the two groups (Table-IV).

**Table-IV:** Comparison of mean magnesium levels in Nephropathic patients with and without diabetes (N=200).

*Nephropathic patients with/ without diabetes mellitus*	*Magnesium levels (mg/dL)*	

	*Mean*	*SD*
Yes	1.54	0.301
No	1.92	0.313

P-value=0.001

Stratification for age shows that out of 51 cases of diabetes in nephropathy 20 were between 25-50 years and 31 were between 51-70 years of age, p value was calculated as 0.475. The gender stratification showed that out of 51 cases of diabetes in nephropathy 27 were male and 24 were females. The p-value was calculated as 0.791. Stratification for duration of DM showed that out of 51 cases of diabetes in nephropathy 20 were between 1-2 years and 31 had >2 years of duration of illness, p-value was calculated as 0.0011.

## DISCUSSION

Diabetic nephropathy is a major health concern. It has caused significant reduction in the life expectancy and quality of life of the diabetic patients.^13^ It is recommended by the American Diabetes Association (ADA) that all Type-2 diabetic patients should undergo urine test for proteinuria, starting at the time of diagnosis and on annual basis.^14^ A prospective diabetes study in United Kingdom reveals that the prevalence rate of nephropathy in diabetic patients (Type-2) was 30.8%, while it was 31% in Mexican Americans.^15^ Along with that studies conducted in Asian region reported variations in the prevalence rate of micro albuminuria ranging from 14.2%, 24.2% and 36.3% in Iran, Pakistan and India respectively. While, the prevalence of macroalbuminuria was 12.7% in Taiwan and 11.2% in Thailand. Microalbuminuria prevalence in European countries was 26.9% in Hungary, while 16% in Italy and 9% in Germany.^13^

In this study, we determined the frequency of diabetes mellitus in patients with nephropathy and compared mean magnesium levels in nephropathic patients with and without diabetes so that we can use this cheap and easily available test as a predictor for the development of nephropathy and its progression in Type 2 diabetic patients. In our study the frequency of diabetes mellitus in patients with nephropathy was 25.5%. Diagnosing diabetes in nephropathic patients and its effective management can not only decrease the morbidity associated with it but also halt the progression of nephropathy.

Al-Rubeaan and colleagues did a study on 54,670 patients with Type-2 diabetes aged ≥ 25 years and concluded that risk factors for macroalbuminuria and microalbuminuria are hypertension, retinopathy, hyperlipidemia, neuropathy and obesity, while diabetes duration, male gender and age greater than 45 years are risk factors for macroalbuminuria only.^16^

For all nephropathic patients having Type-2 diabetes, management includes glycemic and hypertensive control with renin angiotensin aldosterone system inhibitors (RAASI) along with lowering of protein intake, lipid control, dietary salt restriction, increased physical activity, smoking cessation and weight reduction. All these measures can reduce the progression of nephropathy and the risk for cardiovascular events. Good glycemic control prevents and improves microvascular complications. The efficacy of glycemic control as a renoprotective strategy depends on the stage at which it is begun and also depends on the degree of normalization of glucose metabolism. Glycemic control can partially reverse early glomerular hyperfiltration and new-onset microalbuminuria. Glycemic control can also stabilize and/or retard progression in diabetic patients with overt nephropathy.^17^

The Kumamoto study done by Scichiri and associates reported a 60% reduction in proteinuria in young Type-2 diabetic patients achieving an HbA1c of 7.0%.^18^ The authors of the United Kingdom Prospective Diabetes Study (UKPDS) in an outcome and cost-effective analysis, stated that intensive blood glucose control in Type-2 DM patients significantly increased treatment costs and markedly reduced the cost of management of complications.^15^

In our study more than one-fourth (1/4th) patients of nephropathy were diabetic. Early diagnosis and timely management of diabetes can halt the progress of nephropathy.

In the human body Magnesium (Mg) is the 4th most abundant cation and have an important role in basic biological processes. Magnesium deficiency is often related with poor glycemic control. Mg supplementation improves the insulin sensitivity.^5^

The results of the study conducted by Qurrat-ul-Ain et al. showed that serum Mg has significant inverse correlation with Fasting plasma glucose (r=-0.543, *p=*0.001) and Albumin Creatinine Ratio (r=-0.474; *p=*0.001). Mean serum Mg was 0.78 mmol/l in hyperglycemics and 0.88 mmol/l in normoglycemics (*p=*0.001). In Type-2 Diabetes Mellitus, the frequency of hypomagnesaemia patients was 18.8% while no patient with normoglycemia and pre-diabetes showed hypomagnesemia. They conclude that subjects with hyperglycemia had significantly lower mean serum Magnesium levels compared with healthy patients. Hypomagnesemia was also associated with diabetic nephropathy and poor glycemic control.^12^ These findings are consistent with the findings with our study i.e. nephropathic patients having diabetes had 1.54±0.301 mg/dL magnesium levels while cases without diabetes had 1.92±0.313 mg/dL levels of magnesium (p-value=0.001).

A study done on Chinese population by Jiancheng et al. signified the association of Magnesium levels in the serum of the patients with diabetes with and without complications. The serum Magnesium levels in the patients with impaired glucose tolerance (IGT), impaired fasting glucose tolerance (IFGT), Type-1 diabetes (T1D) and Type-2 diabetes (T2D) were found to be significantly lower than that of the control patients. The magnesium levels in the urine were significantly increased only in Type-2 Diabetes and Type-1 Diabetes patients as compared to control group. Type-2 diabetic patients with nephropathy have lower magnesium levels as compared to non-diabetic nephropathic patients.^19^

Mg supplementation can be renoprotective in diabetic nephropathy. Reduction of risk associated with Type-2 diabetes is associated with higher intake of Mg.^20^ Magnesium chloride (MgCl_2_) supplementation lowers HBA1c levels in Type-2 diabetes and improves the insulin resistance index.^21^ In a few studies conducted on animals the effect of Mg on renal function was studied. Parvizi and his associates in a rat model showed that diabetic nephropathy was associated with oxidative stress and high blood glucose level and control of hyperglycemia using Magnesium supplementation can reduce oxidative damages and improve renal dysfunction by lowering of creatinine and blood urea nitrogen (BUN).^22^ The effect of Magnesium supplementation on diabetic nephropathy, however, has not been directly studied.

Hypomagnesaemia patients have an increased risk for diabetes complications and show a more rapid disease progression. Insulin resistance decreases renal Mg2+ reabsorption that results in excretion of urinary Mg2+ which leads to development of a cycle between hypomagnesaemia and insulin resistance. Dietary Mg2+ supplementation for patients with T2DM improves insulin sensitivity and glucose metabolism. In addition to all these, patients with diabetic neuropathy having episodes of diarrhea may suffer from intestinal malabsorption of Mg^2+^, another risk factor for hypomagnesemia.^21^

Our findings taken together signified that hypomagnesemia in patients with diabetic nephropathy may be an independent predictor of the disease progression. The persistent hypomagnesaemia leads to increased serum glucose level, the degree of magnesium depletion and insulin resistance correlates positively with serum glucose concentration and the degree of glycosuria that can lead to sever morbidity and mortality. However, results of this study will helps us to give timely treatment to control magnesium levels and halt the progression of nephropathy as we find decreased mean magnesium levels in Type-2 diabetic nephropathic patients.

## CONCLUSION

The frequency of diabetes mellitus is higher in patients with nephropathy while on comparison of mean magnesium levels; nephropathic patients with diabetes had significant lower levels of magnesium as compared to without diabetes. This suggests that periodic monitoring of Mg levels in diabetic patients may be helpful in early detection and better management of diabetic nephropathy.

### Authors’ Contribution

**MA** conceived and designed the study.

**AI and NFB** did data collection and manuscript writing.

**FAR and UM** did statistical analysis & editing of manuscript.

**NFB** did review and final approval of manuscript.
